# Supplementary material for: Suppression of heterotopic ossification in fibrodysplasia ossificans progressiva using AAV gene delivery
Source: Nat Commun. 2022 Oct 19;13:6175. doi: 10.1038/s41467-022-33956-9 (PMC9579182; doi:10.1038/s41467-022-33956-9)
Supplement: Supplementary file 1 — Supplementary information [file 41467_2022_33956_MOESM1_ESM.pdf]

Suppression of Heterotopic Ossification in Fibrodysplasia Ossificans Progressiva Using AAV Gene Delivery

Yeon-Suk Yang, Jung-Min Kim, Jun Xie, Sachin Chaugule, Chujiao Lin, Hong Ma, Edward Hsiao, Jaehyoung Hong, Hyonho Chun, Eileen M. Shore, Frederick S. Kaplan, Guangping Gao, and Jae-Hyuck Shim

Supplementary information

- Supplementary Figures 1-13
- Supplementary Table 1

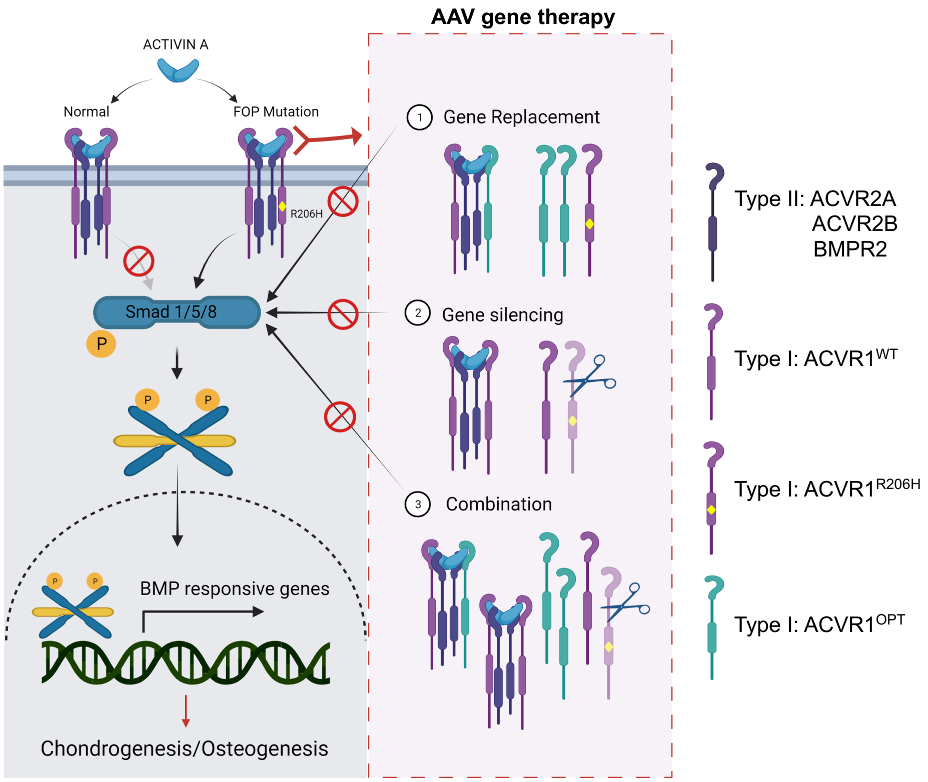

Supplementary Figure 1: Schematic diagram showing molecular mechanisms by which AAV gene therapy suppresses Activin A-induced aberrant BMP signaling by the ACVR1^{R206H} receptor (created with biorender.com)

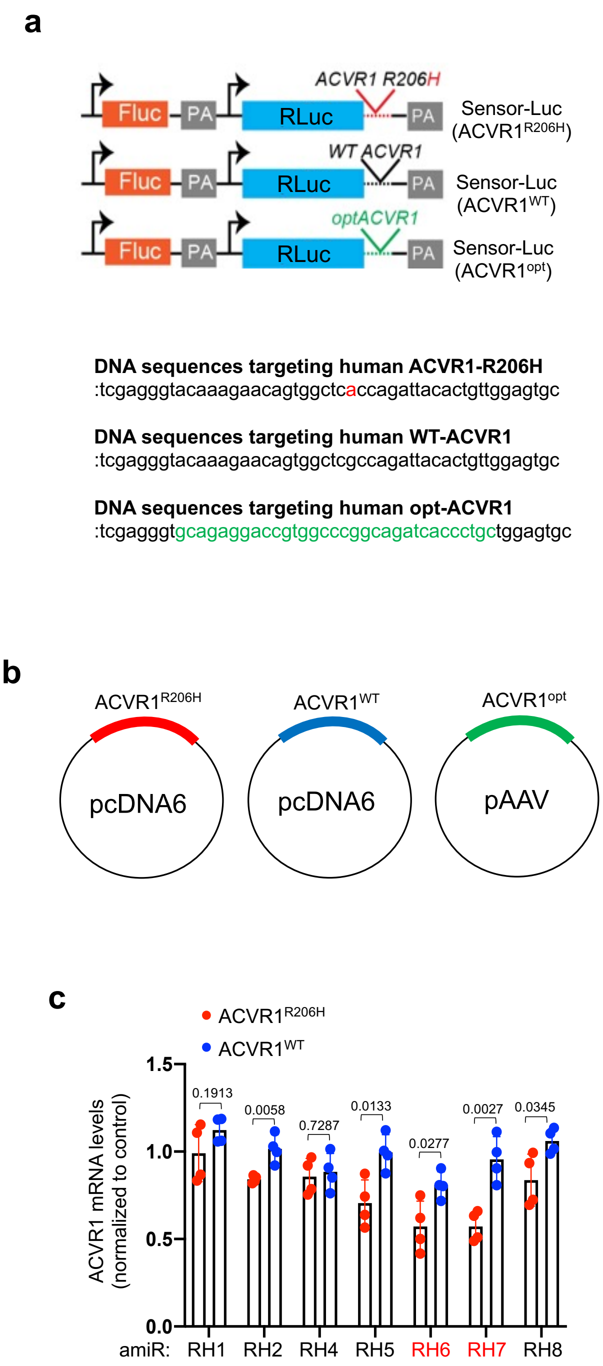

Supplementary Figure 2: Generation of AAV vector genome for gene replacement, silencing, and the combination. a. Schematic diagram of the amiR-sensor plasmids (sensor-Luc) that contain Firefly luciferase (Fluc), *Renilla* luciferase (RLuc), poly A tail (PA), and complimentary sequences of *amiRs* targeting human $ACVR1^{R206H}$, $ACVR1^{WT}$, or $ACVR1^{opt}$. Fluc is used to measure transfection efficiency. **b.** Schematic diagram of the mammalian expression vectors (pcDNA6, pAAV) that encode human $ACVR1^{R206H}$, $ACVR1^{WT}$, or $ACVR1^{opt}$ cDNA. **c.** Plasmids encoding *amiR-ctrl* or *amiRs* specific for human $ACVR1^{R206H}$ were transiently transfected into HEK293 cells along with a plasmid expressing human $ACVR1^{R206H}$ or $ACVR1^{WT}$ cDNA. mRNA levels of $ACVR1^{WT}$ or $ACVR1^{R206H}$ were assessed by RT-PCR and normalized to *amiR-ctrl* (*n*=4). Values represent mean ± SD by an unpaired two-tailed Student's *t*-test (**c**).

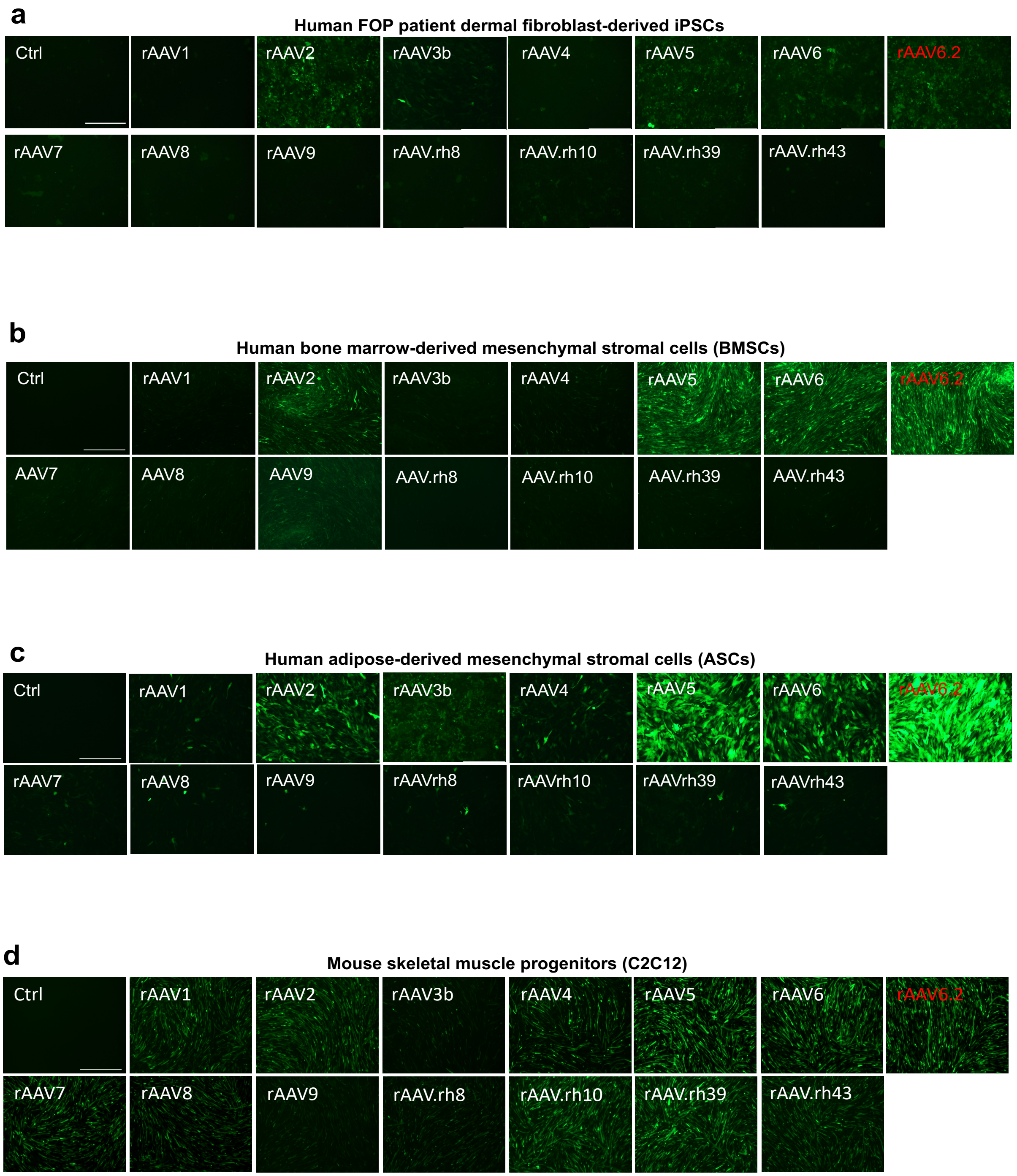

Supplementary Figure 3: Identification of rAAV serotypes transducing human and mouse osteogenic cells *in vitro*. Human FOP patient dermal fibroblast-derived iPSCs (**a**), human bone marrow-derived mesenchymal stromal cells (BMSCs, **b**), human adipose-derived stromal cells (ASCs, **c**), or mouse skeletal muscle progenitors (C2C12, **d**) were treated with PBS or 5 x 10^{10} genome copies (GCs) of 15 different AAV capsids packaged with the same *CBA-Egfp* transgene. Two days later, EGFP expression was assessed by fluorescence microscopy. Alternatively, immunoblotting analysis for EGFP was performed (**Figure 2a**). Scale bars: 500 μm. Data are representative of three independent experiments.

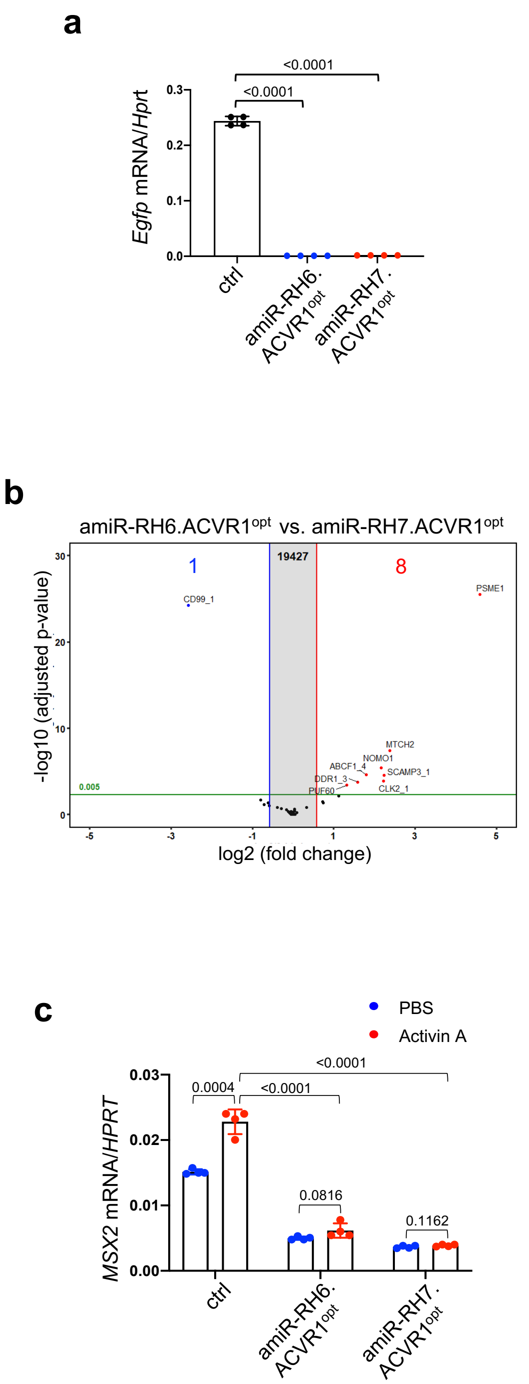

Supplementary Figure 4: Characterization of rAAV-treated human FOP iPSCs. 5 x 10^{10} GCs of AAV6.2 carrying EGFP control, *amiR-RH6.ACVR1^{opt}*, or *amiR-RH7.ACVR1^{opt}* were transduced to human FOP-iPSCs and cultured under osteogenic conditions for four days. Total RNA was subjected to cDNAs synthesis, followed by RT-PCR for EGFP expression (**a**, *n*=4). Alternatively, total RNA was subjected to RNA sequencing. A volcano plot comparing the gene expression for up/downregulated genes in the cells expressing *amiR-RH7.ACVR1^{opt}* relative to *amiR-RH6.ACVR1^{opt}* is displayed (**b,** *n*=2). A volcano plot was generated from multiple t-test. AAV-treated cells were incubated with PBS or Activin A (100 ng/ml) for 12 hours, and *MSX2* mRNA levels were measured by RT-PCR and normalized to *HPRT* (**c**, *n*=4). Values represent mean ± SD by one-way ANOVA test (**a, c**).

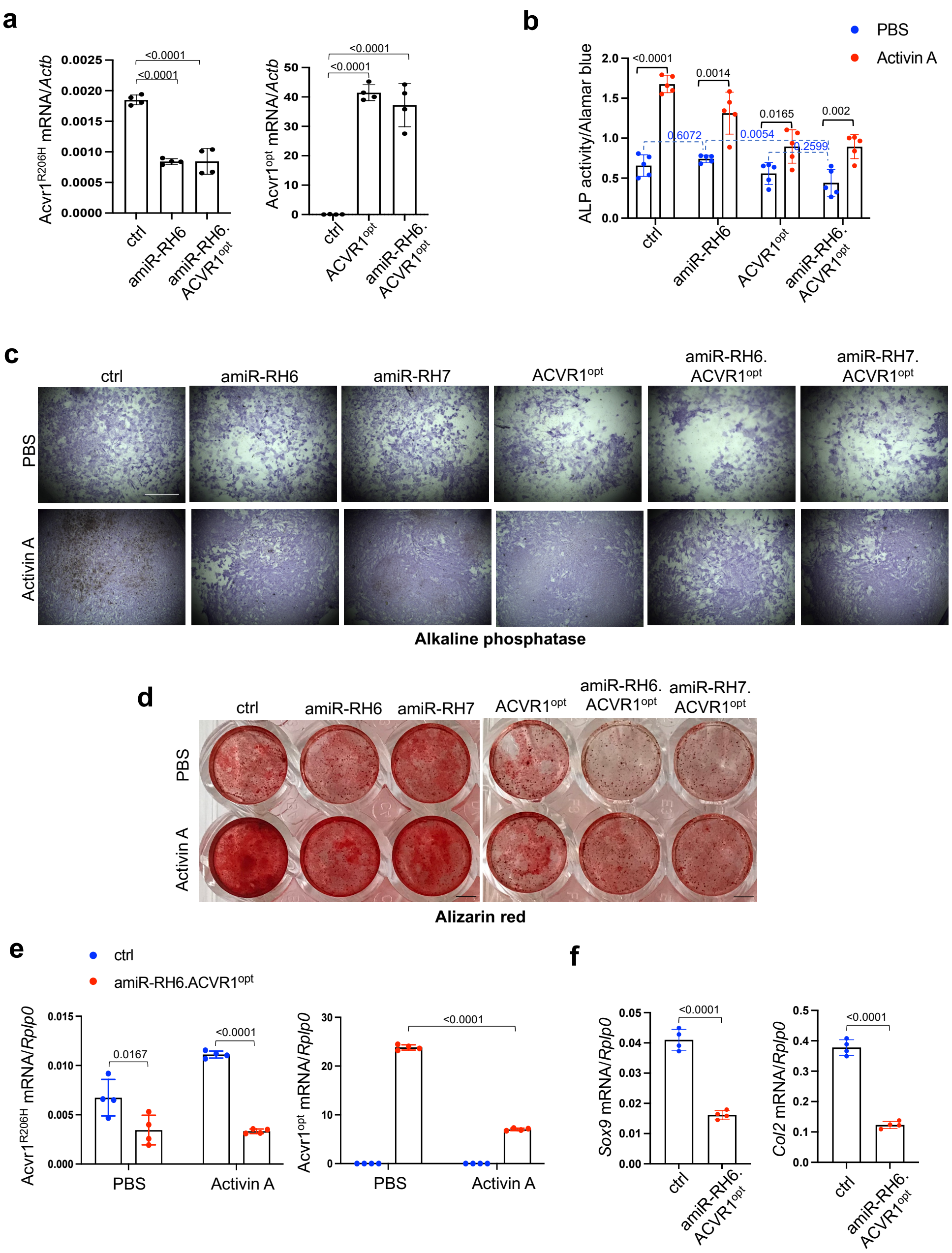

Supplementary Figure 5: AAV gene therapy inhibits Activin A-induced osteogenesis and chondrogenesis of FOP cells. a–d. Osteogenic progenitors were isolated from 4-week-old male *Acvr1*$^{(R206H)Fl}$*;PRRX1-cre* femurs and treated with 5 x 10^{10} GCs of AAV6.2 carrying EGFP control*, amiR-RH6, ACVR1*^{opt}, or *amiR-RH6.ACVR1*^{opt}. Two days later, mRNA levels of *ACVR1*^{R206H} and *ACVR1*^{opt} were measured by RT-PCR (**a**, *n*=4). AAV-treated cells were cultured under osteogenic conditions with PBS or Activin A (50 ng/ml), and ALP activity (**b**, *n*=5) and staining (**c**) and alizarin red staining (**d**) were performed to assess osteoblast differentiation at 6 and 12 days of osteogenic culture, respectively. Alamar blue staining was used for cell viability. **e-f.** Chondrogenic progenitors were isolated from the knee joints of P2 *Acvr1*$^{(R206H)Fl}$*;PRRX1-cre* neonates, treated with 5 x 10^{10} GCs of AAV6.2 carrying EGFP control or *amiR-RH6.ACVR1*^{opt}, and cultured under chondrogenic conditions for four days. mRNA levels of *ACVR1*^{R206H}, *ACVR1*^{opt} (**e**, *n*=4**)**, *Sox9*, and *Col2a1* (**f**, *n*=4**)** were measured by RT-PCR. Scale bars: 200 µm, **c**; 1 mm, **d**. Values represent mean ± SD by an unpaired two-tailed Student's *t*-test (**e, f**) or one-way ANOVA test (**a, b**).

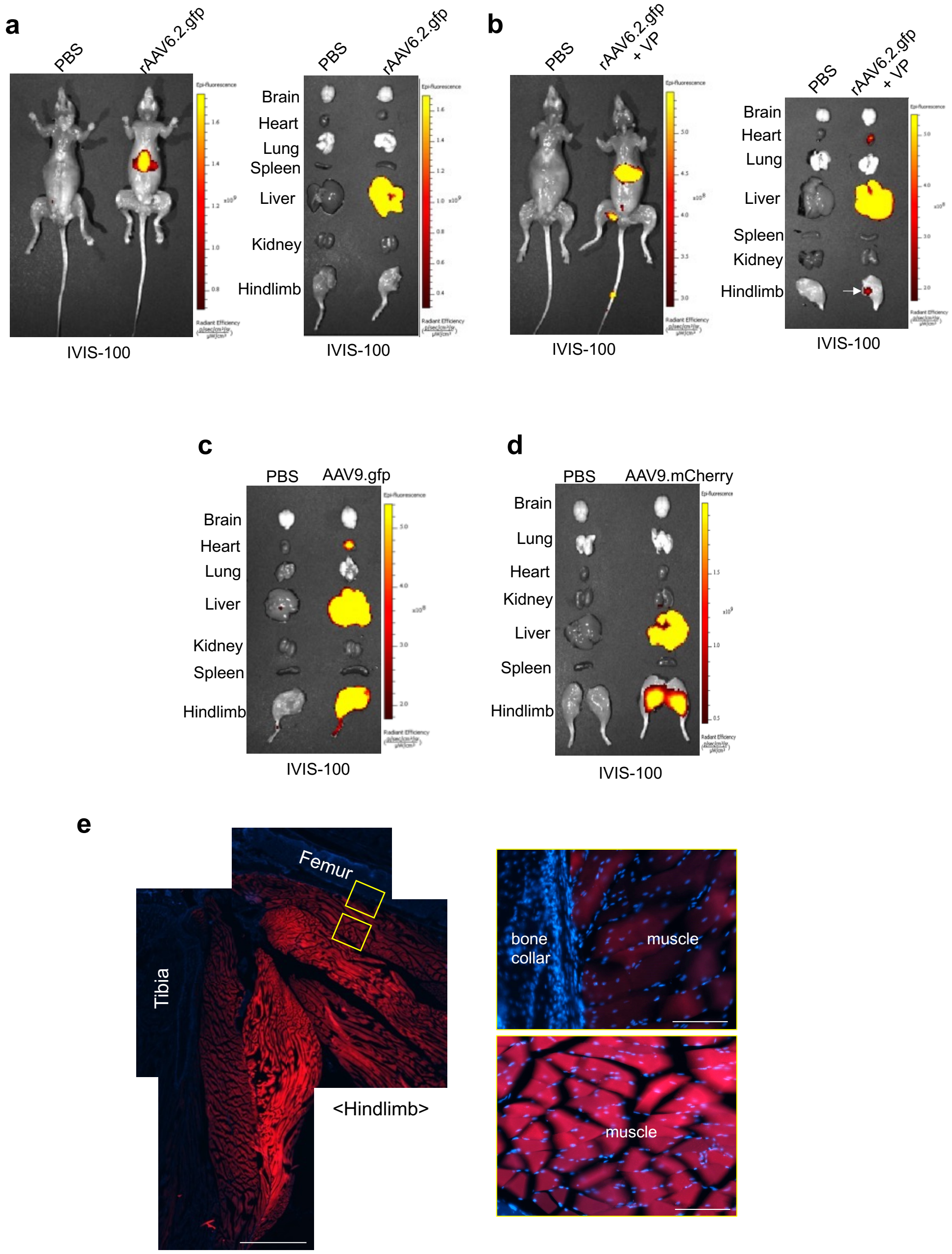

Supplementary Figure 6: Biodistribution of systemically or locally delivered rAAV6.2 or rAAV9 in mice. a, b. 5 x 10^{13} vg/kg of rAAV6.2.*egfp* alone (**a**) or together with vascular permeability agents (VP, human VEGF-166 + sodium heparin + serum albumin (**b**) was intravenously (i.v.) injected into 2-month-old mice (*n* = 3), and two weeks later, the whole body and individual tissue distribution of vectors were assessed by EGFP expression using the IVIS-100 optical imaging system. **c.** 5 x 10^{13} vg/kg of rAAV9.*egfp* was i.v. injected into 2-month-old mice (*n* = 3), and two weeks later, individual tissue distribution of vectors was assessed by EGFP expression using IVIS-100 optical imaging system. **d, e.** 5 x 10^{12} vg/kg of rAAV9.*mCherry* was transdermally (t.d.) injected into the hindlimbs of 2-month-old mice (*n* = 3). Two weeks later, the individual tissue distribution of vectors was assessed by mCherry expression using the IVIS-100 optical imaging system (**d**) or histology on a frozen section of AAV-treated hindlimbs (**e**). DAPI was used for nuclear staining. Scale bars: 1 mm, **left**; 200 μm, **right**.

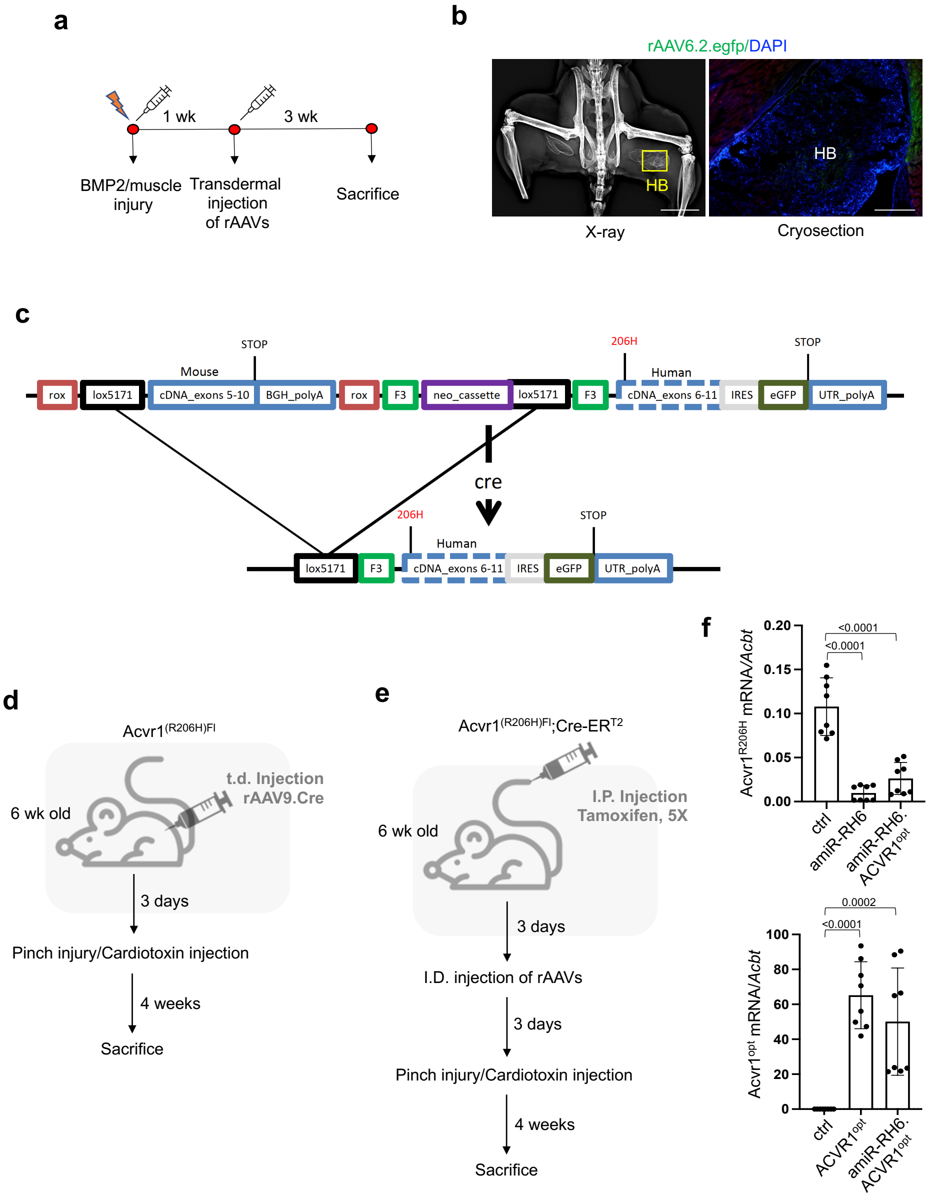

Supplementary Figure 7: Effects of rAAVs on trauma-induced HO in the skeletal muscle. a. Diagram of the study and treatment methods. **b.** 5 x 10^{12} vg/kg of rAAV6.2.*egfp* was t.d. injected into the quadriceps of 2-month-old male mice (n = 3) one week after rBMP2/7/matrigel injection and muscle injury. Three weeks later, radiography of hindlimbs (**b, left**) and histology on a frozen section of HO lesions in the muscle (**b, right**) were performed. The yellow box indicates heterotopic bone (HB) in the skeletal muscle. Scale bars: 5 mm, **left**; 400 µm, **right**. **c.** Diagram showing the targeting construct to generate mice harboring a conditional $Acvr1^{R206H}$ knock-in allele ($Acvr1^{(R206H)Fl}$). **d.** Diagram of the study and treatment methods for **Figure 3f and g**. **e.** Diagram of the study and treatment methods for **Figure 3h**. **f.** 5 x 10^{12} vg/kg of rAAV9 carrying EGFP control, *amiR-RH6*, $ACVR1^{opt}$, or *amiR-RH6.*$ACVR1^{opt}$ was t.d. injected into the hindlimbs of 6-week-old $Acvr1R^{(206H)Fl}$;$Cre\text{-}ER^{T2}$ mice (n = 8) three days after the mice had been intraperitoneally (i.p.) injected 5 times with tamoxifen (10 mg/kg). Four weeks after a 1 µM cardiotoxin/pinch injury was introduced to the gastrocnemius muscle, $Acvr1^{R206H}$ and $ACVR1^{opt}$ mRNA levels were measured by RT-PCR**.** The same experiments were performed in **Figure 3i and j**. Values represent mean ± SD by one-way ANOVA test (**f**). Diagrams in **a**, **d**, and **e** were created with biorender.com.

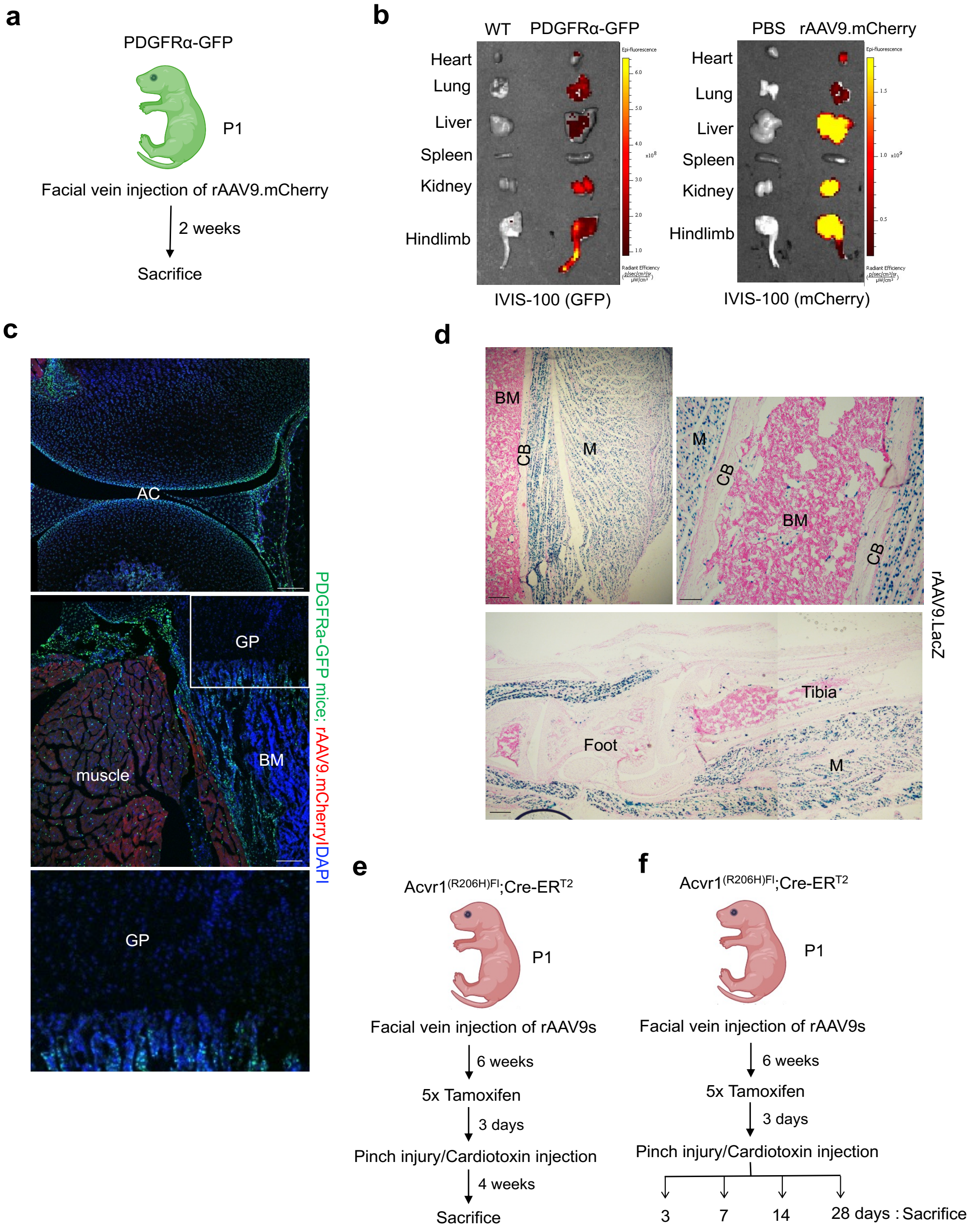

Supplementary Figure 8: Biodistribution of systemically delivered rAAV9 at birth in mice. a. Diagram of the study and treatment methods for **Figure 4a**. **b-c.** P1 *PDGFRα-GFP* neonates (n = 3) were i.v. injected with 10^{11} GCs of rAAV9.*mCherry*, and two weeks later, PDGFRα expression and AAV-transduced tissues were assessed by GFP and mCherry expression, respectively, using the IVIS-100 optical imaging system (**b**). A frozen section of tibias shows AAV-transduced cells by mCherry expression using fluorescence microscopy (**c**). AC: articular cartilage, GP: growth plate, BM: bone marrow. Scale bars: 100 µm. **d.** P1 wildtype neonates (n = 3) were i.v. injected with 10^{11} GCs of rAAV9.*LacZ*, and two weeks later, frozen sections of AAV-transduced tissues were stained for β-galactosidase. CB: cortical bone, M: muscle, BM: bone marrow. Scale bars: 100 µm. **e.** Diagram of the study and treatment methods for **Figure 4b-d**. **e.** Diagram of the study and treatment methods for **Figure 4d**. **f.** Diagram of the study and treatment methods for **Figure 4e and f**. Diagrams in **a**, **e**, and **f** were created with biorender.com. Data are representative images of three independent experiments (**c**, **d**).

a

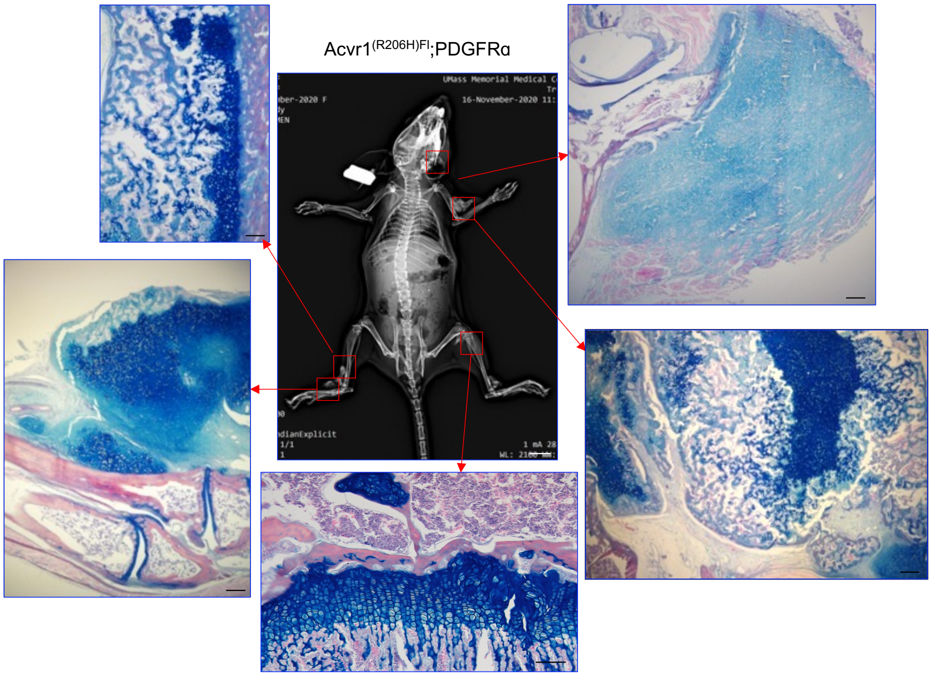

b

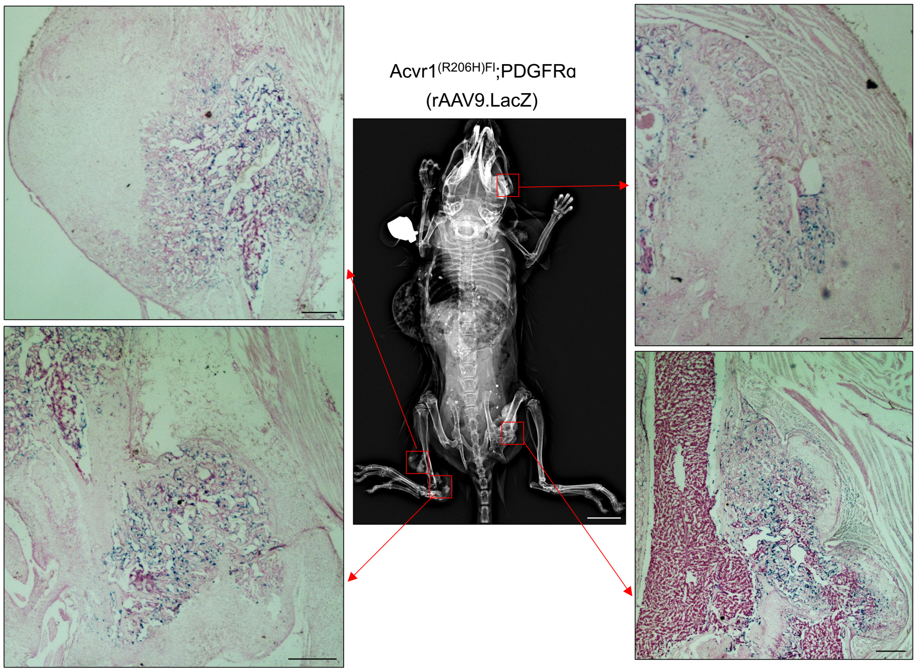

Supplementary Figure 9: Systemic delivery of rAAV9 at birth transduces HO lesions in FOP mice. a. Radiography of the whole body and Alcian blue staining of HO lesions at multiple anatomical locations, including jaws, forearms, hindlimbs, and ankles and knees, in 5-week-old male *Acvr1R$^{(206H)Fl}$;PDGFRα-cre mice* (*n* = 3). Scale bars: 5 mm, **X-ray**; 100 μm, **histology**. **b.** 5 x 10^{13} vg/kg of rAAV9.*LacZ* was i.v. injected into 3-week-old male *Acvr1R$^{(206H)Fl}$;PDGFRα-cre mice* (*n* = 3). Two weeks later, radiography of the whole body was performed to locate HO lesions, and frozen sections of HO tissues were stained for β-galactosidase. These results represent lower magnifications of the images in **Figure 5a**, **bottom**. Scale bars: 5 mm, **X-ray**; 100 μm, **histology**.

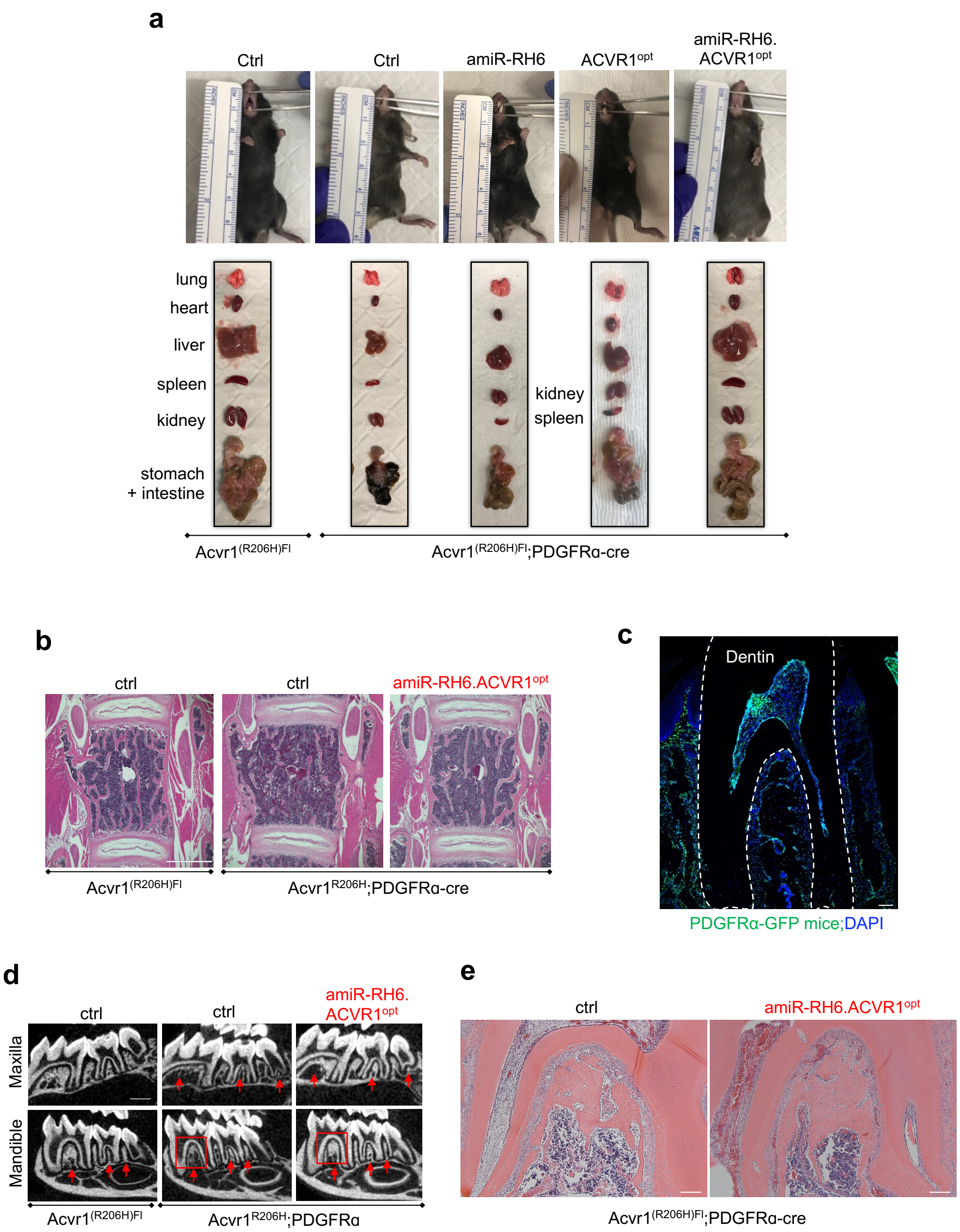

Supplementary Figure 10: Characterization of HO phenotypes in FOP mice after systemic delivery of rAAVs at birth. a, b. P1 *Acvr1*$^{(R206H)Fl}$ or *Acvr1*$^{(R206H)Fl}$*;PDGFR*α-cre neonates were i.v. injected with 10^{11} GCs of rAAV9 carrying EGFP control, *amiR-RH6*, *ACVR1*^{opt}, or *amiR-RH6.ACVR1*^{opt}. Five weeks later, the distance of open mouth (**a, top**) and internal organs (**a, bottom**) of AAV-treated mice were assessed by photography (n = 12). EGFP control-treated *Acvr1*$^{(R206H)Fl}$*;PDGFR*α-cre mice show a decay of intestines due to starvation. H&E staining of the longitudinal sections of lumbar vertebrae (L4) was performed (**b**). **c.** A frozen section of mandibular bones of 2-month-old female *PDGFR*α*-GFP* mice (n = 3) was performed to visualize PDGFRα expression in the root of alveolar bone using fluorescence microscopy. DAPI: nucleus staining. **d, e.** MicroCT analysis of 2D sagittal section images showing maxillary and mandibular bones of AAV-treated mice (**d**). The arrows indicate roots of alveolar bones in the teeth. The red boxes indicate the alveolar bones for histology (**e**). Scale bars: 1 mm, **b, d**; 100 μm, **c, e**. Data are representative images of three independent experiments (**e**).

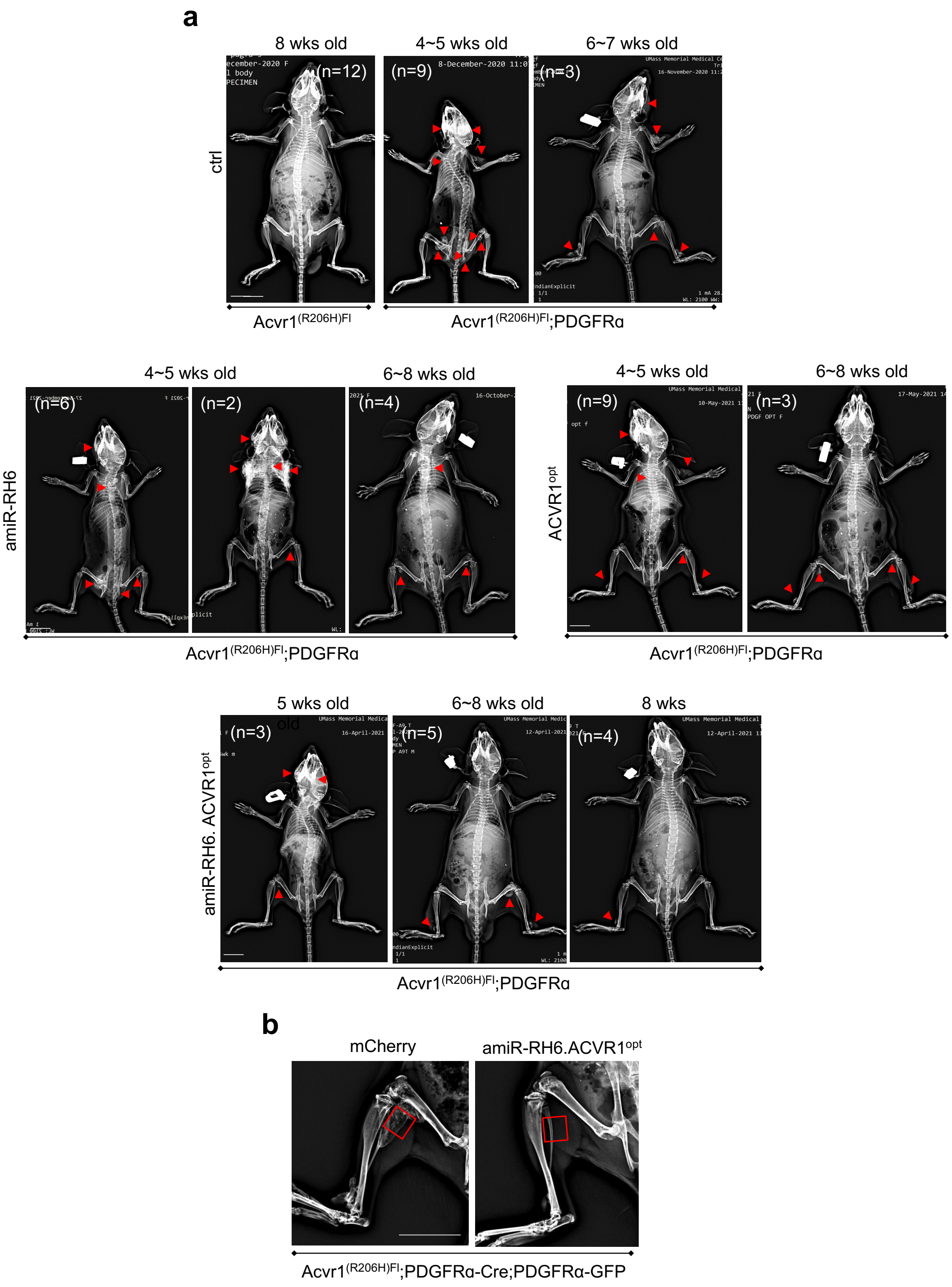

Supplementary Figure 11: Systemic delivery of rAAVs at birth ameliorates spontaneous HO in FOP mice. a. P1 *Acvr1$^{(R206H)Fl}$* or *Acvr1$^{(R206H)Fl}$;PDGFRα*-cre neonates were i.v. injected with 10^{11} GCs of rAAV9 carrying EGFP control, *amiR-RH6*, *ACVR1^{opt}*, or *amiR-RH6.ACVR1^{opt}* (*n* = 12). Radiography was performed on the whole body of AAV-treated mice at different ages to locate HO lesions. The red arrow heads indicate HO lesions. Scale bars: 5 mm. **b.** P1 neonates from *Acvr1$^{(R206H)Fl}$;PDGFRα-cre;PDGFRα-GFP* reporter breeding were i.v. injected with 10^{11} GCs of rAAV9 carrying *mCherry* or *amiR-RH6.ACVR1^{opt}* (*n* = 3) and five weeks later, HO in the tibial muscle was assessed by radiography. Red boxes indicate the frozen section of HO lesions for **Figure 5k**. Scale bars: 5 mm.

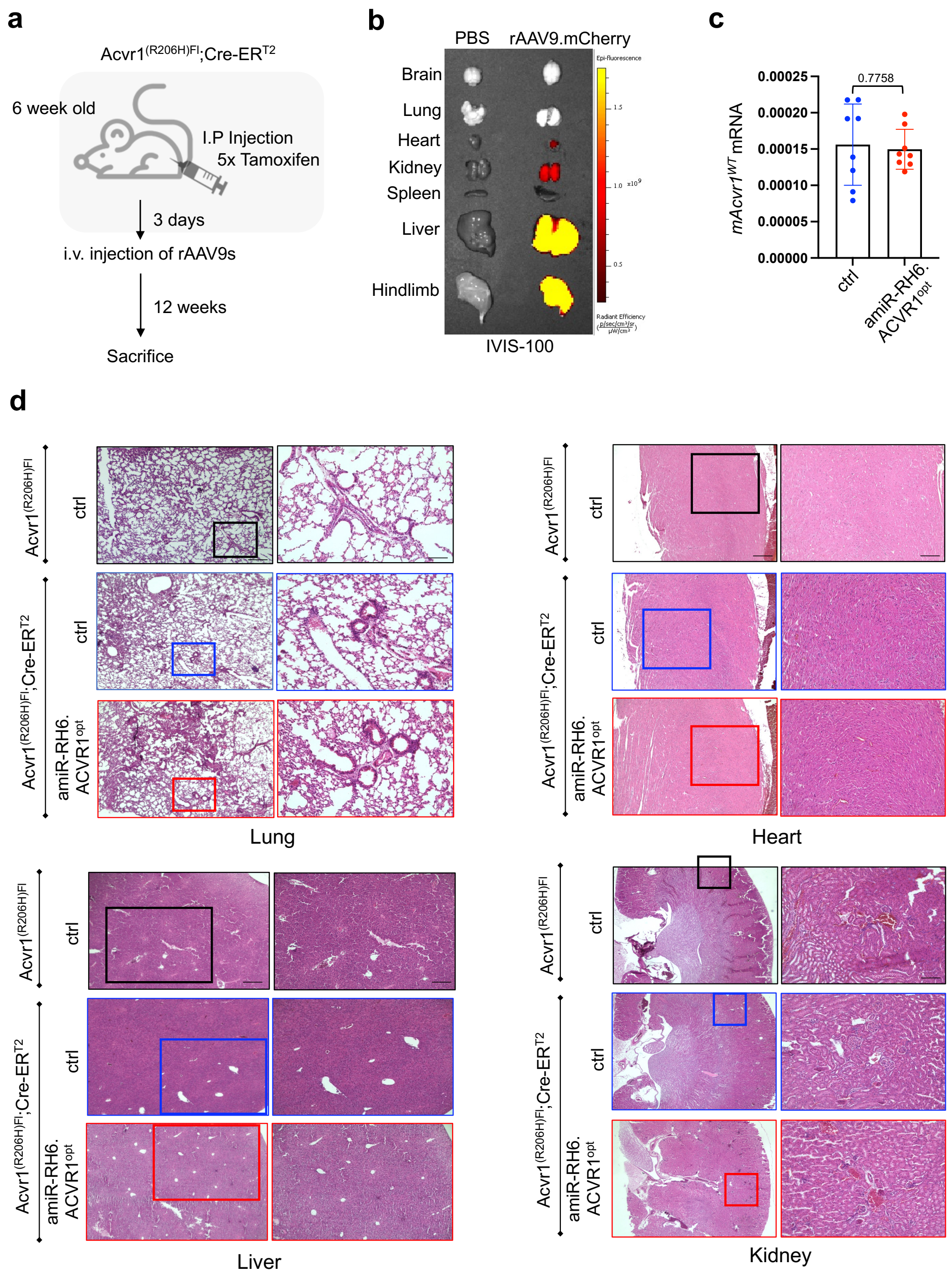

Supplementary Figure 12: Histopathology analysis of non-HO tissues in AAV-treated FOP mice. **a.** Diagram of the study and treatment methods for **Figure 6** (created with biorender.com). **b.** 5 x 10^{13} vg/kg of rAAV9.*mCherry* was i.v. injected into 6-week-old *Acvr1R*$^{(206H)Fl}$*;Cre-ER*^{T2} mice (*n*=3) three days after five times consecutive i.p. injections of tamoxifen (10 mg/kg). mCherry expression in individual tissues was monitored by IVIS-100 optical imaging two weeks post-injection. **c, d.** 5 x 10^{13} vg/kg of EGFP control or *amiR-RH6.ACVR1*^{opt} was i.v. injected into 6-week-old *Acvr1R*$^{(206H)Fl}$ or *Acvr1R*$^{(206H)Fl}$*;Cre-ER*^{T2} mice three days after tamoxifen injection. 12 weeks later, mRNA levels of mouse *Acvr1* in the liver were assessed by RT-PCR (**c,** *n*=8**)** and H&E staining of the longitudinal sections of lung, liver, kidney, and heart was performed to assess tissue histopathology (**d**, *n*=3). Scale bars: 200 µm**, left**; 50 µm, **right**. Values represent mean ± SD by an unpaired two-tailed Student's *t*-test (**c**).

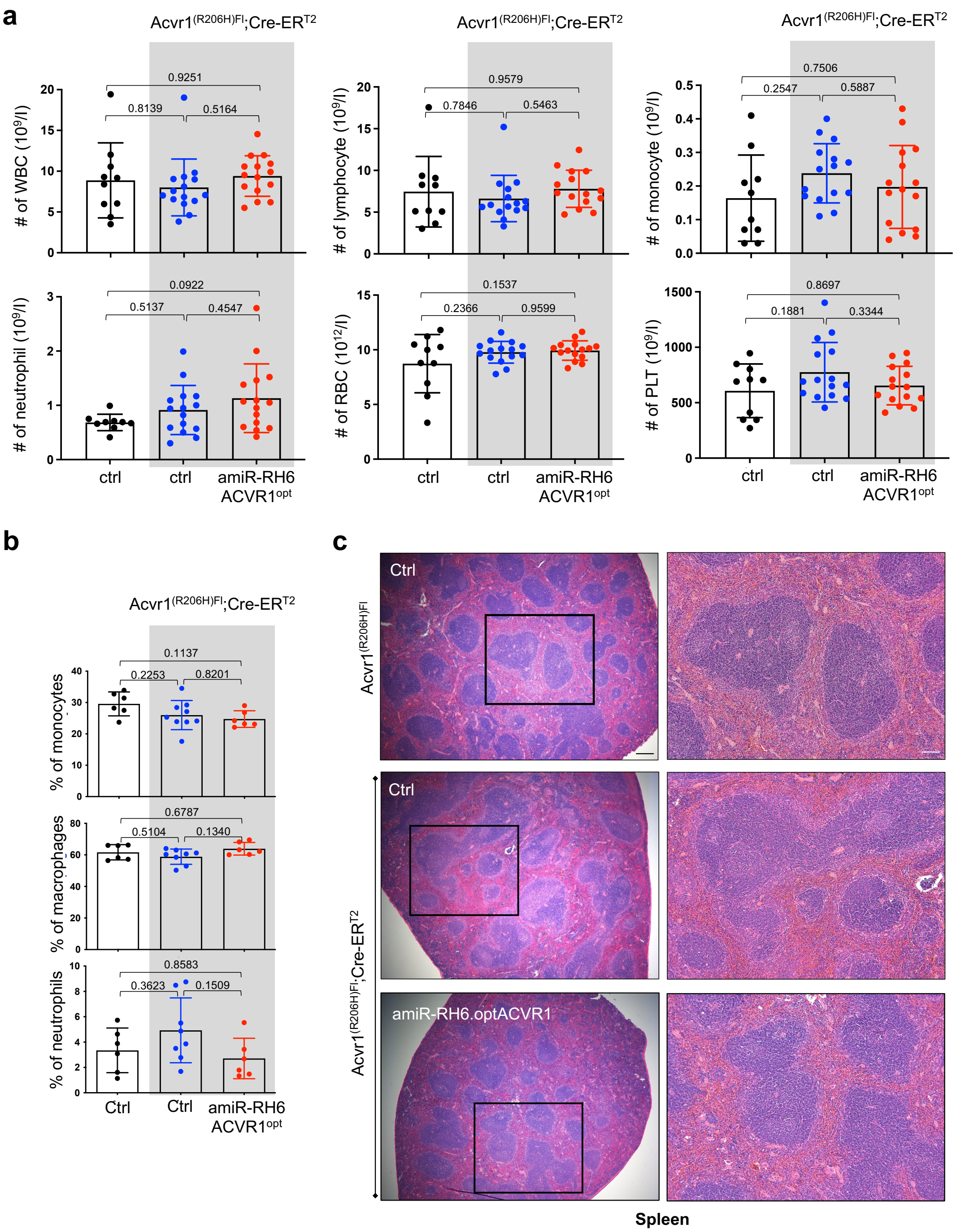

Supplementary Figure 13: No effects of systemically delivered AAV vectors on systemic immunity in FOP mice. a-c. 5 x 10^{13} vg/kg of *EGFP control* or *amiR-RH6.ACVR1^{opt}* was i.v. injected into 6-week-old *Acvr1R$^{(206H)Fl}$* or *Acvr1R$^{(206H)Fl}$;Cre-ER^{T2}* mice three days after tamoxifen injection. 12 weeks later, a complete blood count test was performed to measure numbers of white blood cells (WBC), lymphocytes, monocytes, neutrophils, red blood cells (RBC), and platelets (PLT) in the plasma (**a**, *n*=10–15). Flow cytometry analysis showing the frequency of monocytes, macrophages, and neutrophils within the population of total splenocytes (**b**, *n*=6–8) or H&E staining of the longitudinal sections of the AAV-treated spleens (**c**, *n* = 3) were performed. AAV-treated *Acvr1R$^{(R206H)Fl}$* (WT control) and *Acvr1$^{(R206H)Fl}$;Cre-ER^{T2}* mice (gray boxes) are displayed (**a, b**). Scale bars: 200 μm, **left**; 50 μm, **right**. Values represent mean ± SD by one-way ANOVA test (**a, b**).

Supplementary Table 1: Sequences of primers, probes and gBlocks™

Mouse Genes	Primer (5'-3')	Product length
ID1 (HLH protein)	F: ACATGAACGGCTGCTACTCAC<br>R: GACTTCAGACTCCGAGTTCAGC	143
AIP (Alkaline phosphatase)	F: CACAATATCAAGGATATCGACGTGA<br>R: ACATCAGTTCTGTTCTTCGGGTACA	74
Runx2 *(Runt-related transcription factor 2)*	F: ACATGAACGGCTGCTACTCAC<br>R: GACTTCAGACTCCGAGTTCAGC	143
BSP(IBSP) (Integrin binding sialoprotein)	F: CAGGGAGGCAGTGACTCTTC<br>R: AGTGTGGAAAGTGTGGCGTT	155
Osx (SP7 transcription factor 7)	F: ATGGCGTCCTCTCTGCTTGA<br>R: GAAGGGTGGGTAGTCATTTG	276
OCN / BGLAP (Bone γ-carboxyglutamate protein)	F: GCAGCACAGGTCCTAAATAG<br>R: GGGCAATAAGGTAGTGAACAG	184
HPRT (Hypoxanthine-guanine phosphoribosyl transferase)	F: CTGGTGAAAAGGACCTCTCGAAG<br>R: CCAGTTTCACTAATGACACAAACG	146
ACBT (Actin, Beta)	F: AGGGAAATCGTGCGTGACAT<br>R: GGGCAATAAGGTAGTGAACAG	150
MSX2 (Msh homeobox2)	F: GGAACTGGAAAAGCTGAAAATG<br>R: CACAGGTCTATGGAAGGGGTAG	130
SOX9 (SRY-Box Transcripton Factor9)	F: CACACGTCAAGCGACCCATGAA<br>R: TCTTCTCGCTCTCGTTCAGCAG	147
Col2a1 (Collagen, type II, alpha 1)	F: GCTGGTGAAGAAGGCAAACGAG<br>R: CCATCTTGACCTGGGAATCCAC	113
Acan (Aggrecan)	F: GCCTACCCGGTACCCTACAG<br>R: ACATTGCTCCTGGTCTGCAA	175
RPLP0 (Ribosomal Protein Lateral Stalk Subunit P0)	F: TGGCCAATAAGGTGCCAGCTGCTG<br>R: CTTGTCTCCAGTCTTTATCAGCTGCAC	191
Acvr1 (activin A receptor, type 1)	F: CCATTGAAGGGCTCATCACCAC<br>R: CCGTTCTCTGTACCAGGAAAGG	114
EGFP (Enhanced green fluorescent protein)<br>TaqMan	F: AGCAAAGACCCCAACGAGAA<br>R: GGCGGCGGTCACGAA<br>P: 6FAM-CGCGATCACATGGTCCTGCTGG-TAMRA	
**Human Gene**	**Primer(5'-3')**	**Product length**
**ACVR1**^{R206H}	F: TGGTACAAAGAACAGTGGCTTA<br>R: CCATACCTGCCTTTCCCGA	
**ACVR1**^{OPT}	F: GACTACAAGCCACCCTTCTATG<br>R: ACCAGCGATTAGGGATGTTG	

Gene	DNA sequences
amiR-RH1	gtcttttatttcaggtcccagatctagggctctgcgtttgctccaggtagtccgctgctcccttgggcctgggcccactgacagc cctggtgcctctggccggctgcacacctcctggcgggcagctgtgagtgtaatctggtgagccacttgttctggcaatacctg agtggctctgcggattacactcacggaggcctgccctgactgcccacggtgccgtggccaaagaggatctaagggcacc gctgagggcctacctaaccatcgtggggaataaggacagtgtcacccctgcaggggatccggtggtggtgc
amiR-RH2	agggctctgcgtttgctccaggtagtccgctgctcccttgggcctgggcccactgacagccctggtgcctctggccggctgc acacctcctggcgggcagctgtgagtgtaagctggtgagccacttgttctggcaatacctgagtggctctgcggcttacact cacggaggcctgccctgactgcccacggtgccgtggccaaagaggatctaagggcaccgctgagggcctacctaacc atcgtggggaataaggacagtgtcaccc
amiR-RH3	agggctctgcgtttgctccaggtagtccgctgctcccttgggcctgggcccactgacagccctggtgcctctggccggctgc acacctcctggcgggcagctgtggtgtaatctggtgagccactgtgttctggcaatacctgcagtggctgtctagattacacc acggaggcctgccctgactgcccacggtgccgtggccaaagaggatctaagggcaccgctgagggcctacctaaccat cgtggggaataaggacagtgtcaccc
amiR-RH4	agggctctgcgtttgctccaggtagtccgctgctcccttgggcctgggcccactgacagccctggtgcctctggccggctgc acacctcctggcgggcagctgtggtgtaagctggtgagccactgtgttctggcaatacctgcagtggctgtctagcttacac cacggaggcctgccctgactgcccacggtgccgtggccaaagaggatctaagggcaccgctgagggcctacctaacc atcgtggggaataaggacagtgtcaccc
amiR-RH5	agggctctgcgtttgctccaggtagtccgctgctcccttgggcctgggcccactgacagccctggtgcctctggccggctgc acacctcctggcgggcagctgtgtgtaagctggtgagccactgttgttctggcaatacctgacagtggcagatcagcttaca cacggaggcctgccctgactgcccacggtgccgtggccaaagaggatctaagggcaccgctgagggcctacctaacc atcgtggggaataaggacagtgtcaccc
amiR-RH6	agggctctgcgtttgctccaggtagtccgctgctcccttgggcctgggcccactgacagccctggtgcctctggccggctgc acacctcctggcgggcagctgtgtgtaatctggtgagccactgttgttctggcaatacctgacagtggcagatcagattaca cacggaggcctgccctgactgcccacggtgccgtggccaaagaggatctaagggcaccgctgagggcctacctaacc atcgtggggaataaggacagtgtcaccc
amiR-RH7	agggctctgcgtttgctccaggtagtccgctgctcccttgggcctgggcccactgacagccctggtgcctctggccggctgc acacctcctggcgggcagctgtggtaatctggtgagccactgtttgttctggcaatacctgaacagtgggacgccagattac cacggaggcctgccctgactgcccacggtgccgtggccaaagaggatctaagggcaccgctgagggcctacctaacc atcgtggggaataaggacagtgtcaccc
amiR-RH8	agggctctgcgtttgctccaggtagtccgctgctcccttgggcctgggcccactgacagccctggtgcctctggccggctgc acacctcctggcgggcagctgtggtaagctggtgagccactgtttgttctggcaatacctgaacagtgggacgccagctta ccacggaggcctgccctgactgcccacggtgccgtggccaaagaggatctaagggcaccgctgagggcctacctaac catcgtggggaataaggacagtgtcaccc
amiR-RH9	agggctctgcgtttgctccaggtagtccgctgctcccttgggcctgggcccactgacagccctggtgcctctggccggctgc acacctcctggcgggcagctgtgtaatctggtgagccactgttctgttctggcaatacctggaacagtgcgttaccagattac acggaggcctgccctgactgcccacggtgccgtggccaaagaggatctaagggcaccgctgagggcctacctaaccat cgtggggaataaggacagtgtcaccc
amiR-RH10	agggctctgcgtttgctccaggtagtccgctgctcccttgggcctgggcccactgacagccctggtgcctctggccggctgc acacctcctggcgggcagctgtgtaagctggtgagccactgttctgttctggcaatacctggaacagtgcgttaccagctta cacggaggcctgccctgactgcccacggtgccgtggccaaagaggatctaagggcaccgctgagggcctacctaacc atcgtggggaataaggacagtgtcaccc
amiR-RH11	agggctctgcgtttgctccaggtagtccgctgctcccttgggcctgggcccactgacagccctggtgcctctggccggctgc acacctcctggcgggcagctgtgaatctggtgagccactgttcttgttctggcaatacctgagaacagtcccccaccagatt cacggaggcctgccctgactgcccacggtgccgtggccaaagaggatctaagggcaccgctgagggcctacctaacc atcgtggggaataaggacagtgtcaccc
amiR-RH12	agggctctgcgtttgctccaggtagtccgctgctcccttgggcctgggcccactgacagccctggtgcctctggccggctgc acacctcctggcgggcagctgtgaagctggtgagccactgttcttgttctggcaatacctgagaacagtcccccaccagctt cacggaggcctgccctgactgcccacggtgccgtggccaaagaggatctaagggcaccgctgagggcctacctaacc atcgtggggaataaggacagtgtcaccc
human ACVR1^{OPT} cDNA	atggtcgatggagtgatgatcctgcctgtcctgattatgattgccctgcccagccccagcatggaagatgaaaaacctaaa gtcaaccctaagctgtatatgtgcgtgtgcgagggcctgagctgcggaaacgaggatcactgcgagggccagcagtgttt cagctccctgtccatcaatgacggcttccacgtgtaccagaagggctgctttcaggtgtatgagcagggcaagatgacctg taagacaccaccttccccaggacaggcagtggagtgctgtcagggcgattggtgtaaccggaatatcaccgcccagctg ccaacaaagggcaagtctttccccggcacacagaactttcacctggaagtgggcctgatcatcctgagcgtggtgttcgcc

	gtgtgcctgctggcatgtctgctgggagtggccctgagaaagtttaagcggagaaaccaggagcggctgaatccaagag<br>atgtggagtacggcaccatcgagggcctgatcaccacaaatgtgggcgactctacactggccgacctgctggatcacag<br>ctgcaccagcggctccggatctggcctgccctttctggtgcagaggaccgtggcccggcagatcaccctgctggagtgcg<br>tgggcaagggccggtacggagaagtgtggagaggatcctggcagggagagaacgtggcagtgaagatcttctctagc<br>cgggatgagaagtcttggtttagagagacagagctgtataacacagtgatgctgaggcacgagaatatcctgggcttcat<br>cgcctccgacatgacctctcgccactcctctacacagctgtggctgatcacccactaccacgagatgggctccctgtacga<br>ttacctccagctgaccacactggacacagtgtcttgcctgcggatcgtgctgtctatcgccagcggcctggcacacctgcac<br>atcgagatctttggaacccagggcaagccagcaatcgcacacagagatctgaagtctaagaacatcctggtgaagaag<br>aatggccagtgctgtatcgccgatctgggcctggccgtgatgcacagccagtccaccaaccagctggacgtgggcaaca<br>atcctcgggtgggcacaaagagatacatggccccagaggtgctggatgagacaatccaggtggactgcttcgatagcta<br>taagagggtggacatctgggcctttggcctggtgctgtgggaggtggcaaggaggatggtgagcaacggcatcgtggag<br>gactacaagccacccttctatgacgtggtgcctaatgatccatcctttgaggacatgcgcaaggtggtgtgcgtggatcagc<br>agaggcccaacatccctaatcgctggttcagcgaccccaccctgacatccctggccaagctgatgaaggagtgttggtat<br>cagaatcctagcgccaggctgaccgccctgcgcatcaagaaaactctgactaaaatcgacaatagcctggataaactg<br>aaaaccgactgctga
CBA Intron	gtgagcgggcgggacggcccttctcctccgggctgtaattagcgcttggtttaatgacggcttgtttcttttctgtggctgcgtga<br>aagccttgaggggctccgggagggccctttgtgcggggggagcggctcggggggtgcgtgcgtgtgtgtgtgcgtgggg<br>agcgccgcgtgcggctccgcgctgcccggcggctgtgagcgctgcgggcgcggcgcggggctttgtgcgctccgcagt<br>gtgcgcgaggggagcgcggccgggggcggtgccccgcggtgcggggggggctgcgaggggaacaaaggctgcgt<br>gcggggtgtgtgcgtgggggggtgagcagggggtgtgggcgcgtcggtcgggctgcaaccccccctgcacccccctcc<br>ccgagttgctgagcacggcccggcttcgggtgcggggctccgtacggggcgtggcgcggggctcgccgtgccgggcgg<br>ggggtggcggcaggtgggggtgccgggcggggcggggccgcctcgggccggggagggctcgggggaggggcgcg<br>gcggcccccggagcgccggcggctgtcgaggcgcggcgagccgcagccattgccttttatggtaatcgtgcgagaggg<br>cgcagggacttcctttgtcccaaatctgtgcggagccgaaatctgggaggcgccgccgcaccccctctagcgggcgcgg<br>ggcgaagcggtgcggcgccggcaggaaggaaatgggcggggagggccttcgtgcgtcgccgcgccgccgtccccttc<br>tccctctccagcctcggggctgtccgcgggggggacggctgccttcgggggggacggggcagggcggggttcggcttctg<br>gcgtgtgaccggcggctctagagcctctgctaaccatgttcatgccttcttctttttcctacagctcctgggcaacgtgctggtta<br>ttgtgctgtctcatcattttggcaaag
MBL intron	tcagatcgcctggagacgccatccacgctgttttgacctccatagaagacaccgggaccgatccagcctccgcggccgg<br>gaacggtgcattggaacgcggattccccgtgccaagagtgacgtaagtaccgcctatagagtctataggcccaccccctt<br>ggcttcttatgcatgctatactgtttttggcttggggtctatacacccccgcttcctcatgttTGCTGCCCGTGACCAG<br>CACGTCAACGATTTTGTGGGCACGGGCGACACcgcagtgtagtctgagcagtactcgttgctgccg<br>cgcgcgccaccagacataatagctgacagactaacagactgttcctttccatgggtcttttctgca
Synthetic intron	gaactgaaaaaccagaaagttaactggtaagtttagtctttttgtcttttatttcaggtcccggatccggtggtggtgcaaatca<br>aagaactgctcctcagtggatgttgcctttacttctaggcctgtacggaagtgttacttctgctctaaaagctgcggaattgtac<br>cc
