# Supplementary material for: Suppression of heterotopic ossification in fibrodysplasia ossificans progressiva using AAV gene delivery
Source: Nat Commun. 2022 Oct 19;13:6175. doi: 10.1038/s41467-022-33956-9 (PMC9579182; doi:10.1038/s41467-022-33956-9)
Supplement: Supplementary file 3 — Description of Additional Supplementary Files [file 41467_2022_33956_MOESM3_ESM.pdf]

Suppression of Heterotopic Ossification in Fibrodysplasia Ossificans Progressiva Using AAV Gene Delivery

Supplementary movie 1: P1 *Acvr1*$^{(R206H)Fl}$ or *Acvr1*$^{(R206H)Fl}$*;PDGFR*α neonates were i.v. injected with 10^{11} GCs of rAAV9 carrying *EGFP control* or *amiR-RH6.ACVR1*^{opt} (*n*=12). Movie 1 shows the body size and mobility of 5-week-old male AAV-treated mice, including EGFP control-treated *Acvr1*$^{(R206H)Fl}$ mouse (normal body size and mobility), EGFP control-treated *Acvr1*$^{(R206H)Fl}$*;PDGFR*α-cre mouse (small body size and slow mobility) and *amiR-RH6.ACVR1*^{opt}-treated *Acvr1*$^{(R206H)Fl}$*;PDGFR*α-cre mouse (normal body size and mobility). The arrow indicates the *amiR-RH6.ACVR1*^{opt}-treated *Acvr1*$^{(R206H)Fl}$*;PDGFR*α-cre mouse.

Supplementary movie 2 and 3: 5 x 10^{13} vg/kg of *EGFP control* (**movie 2**) or *amiR-RH6.ACVR1*^{opt} (**movie 3**) was i.v. injected into 6-week-old female *Acvr1R*$^{(206H)Fl}$*;Cre-ER*^{T2} mice (*n*=10) three days after tamoxifen injection. Movie 2 shows the heterotopic bone bridging the femur to the head of the fibula near the knee joints of 16-week-old AAV-treated mice.

Supplementary movie 4: 5 x 10^{13} vg/kg of *EGFP control* or *amiR-RH6.ACVR1*^{opt} was i.v. injected into 6-week-old female *Acvr1R*$^{(206H)Fl}$*;Cre-ER*^{T2} mice (*n*=10) three days after tamoxifen injection. Movie 4 shows the body size and mobility of 16-week-old AAV-treated mice, including an EGFP control-treated *Acvr1*$^{(R206H)Fl}$ mouse (normal mobility and activity level), an EGFP control-treated *Acvr1R*$^{(206H)Fl}$*;Cre-ER*^{T2} mouse (reduced mobility and activity level), and an *amiR-RH6.ACVR1*^{opt}-treated *Acvr1R*$^{(206H)Fl}$*;Cre-ER*^{T2} mouse (normal mobility and activity level). The arrow indicates the *amiR-RH6.ACVR1*^{opt}-treated *Acvr1R*$^{(206H)Fl}$*;Cre-ER*^{T2} mouse.
