# Supplementary figures and images for: Suppression of heterotopic ossification in fibrodysplasia ossificans progressiva using AAV gene delivery
Source: Nat Commun. 2022 Oct 19;13:6175. doi: 10.1038/s41467-022-33956-9 (PMC9579182; doi:10.1038/s41467-022-33956-9)

**Figure 1b**

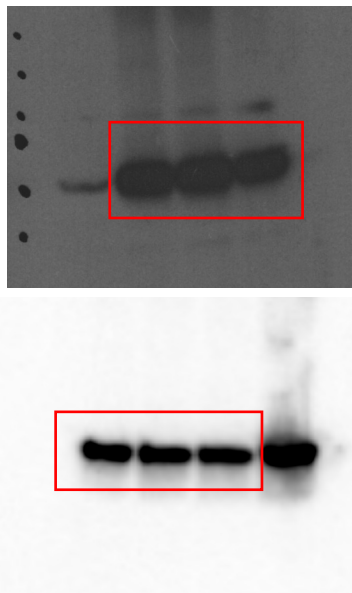

**Figure 1h**

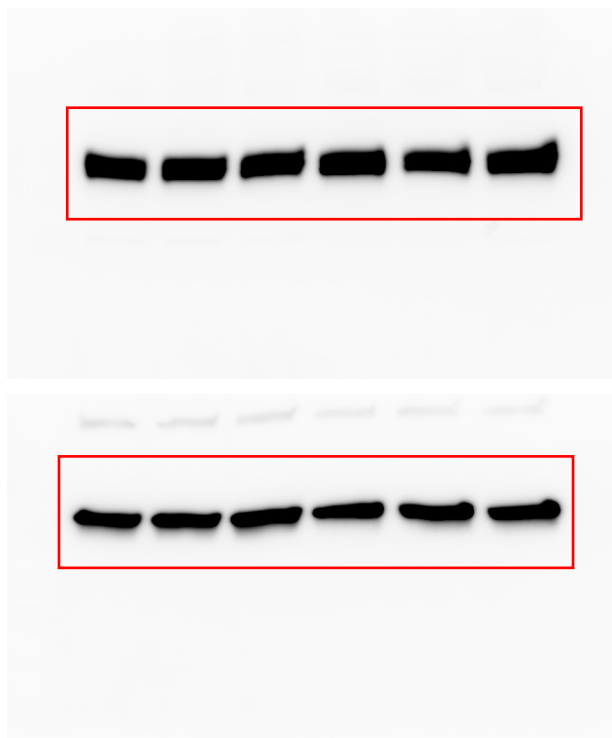

Figure 1e

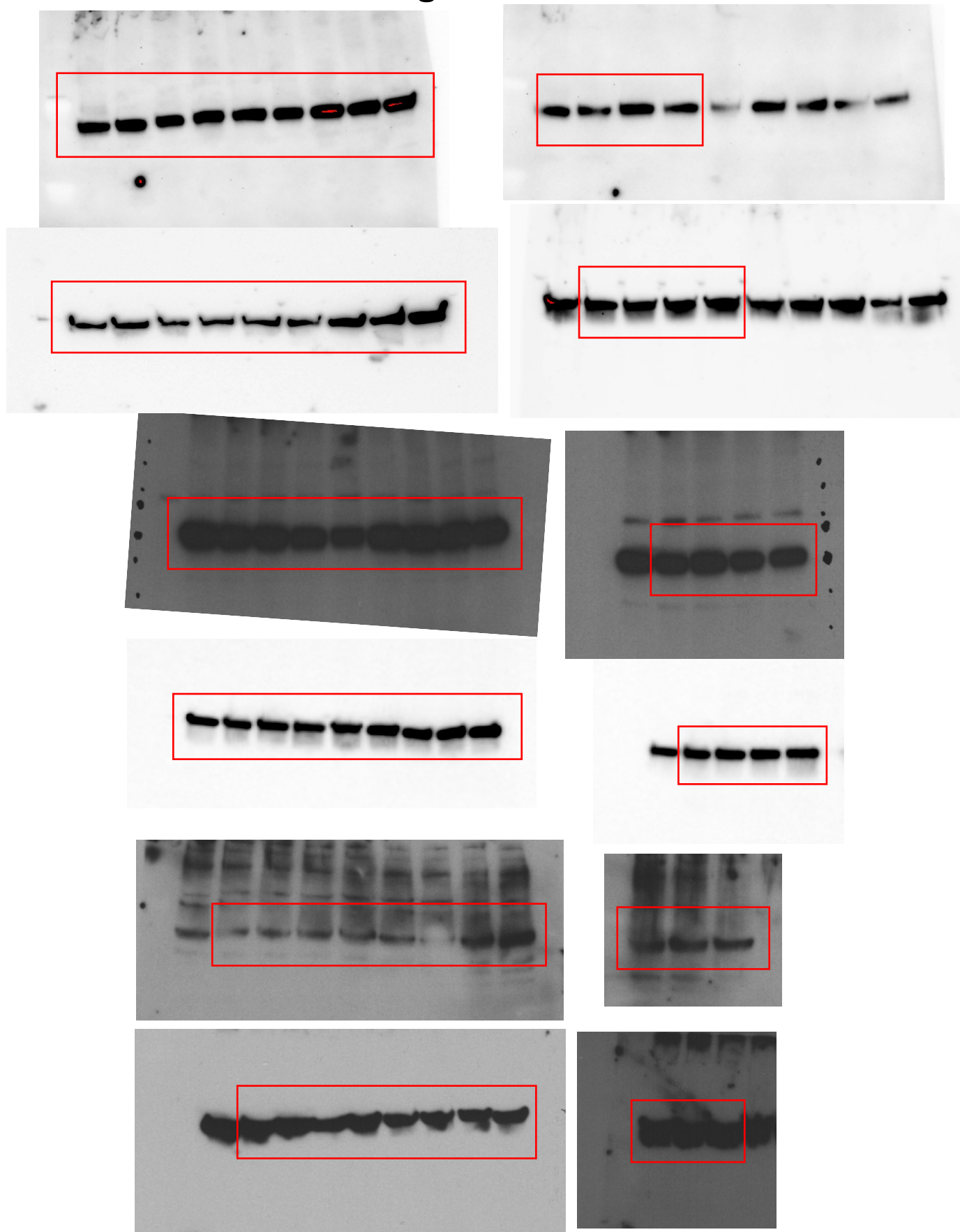

**Figure 2a**

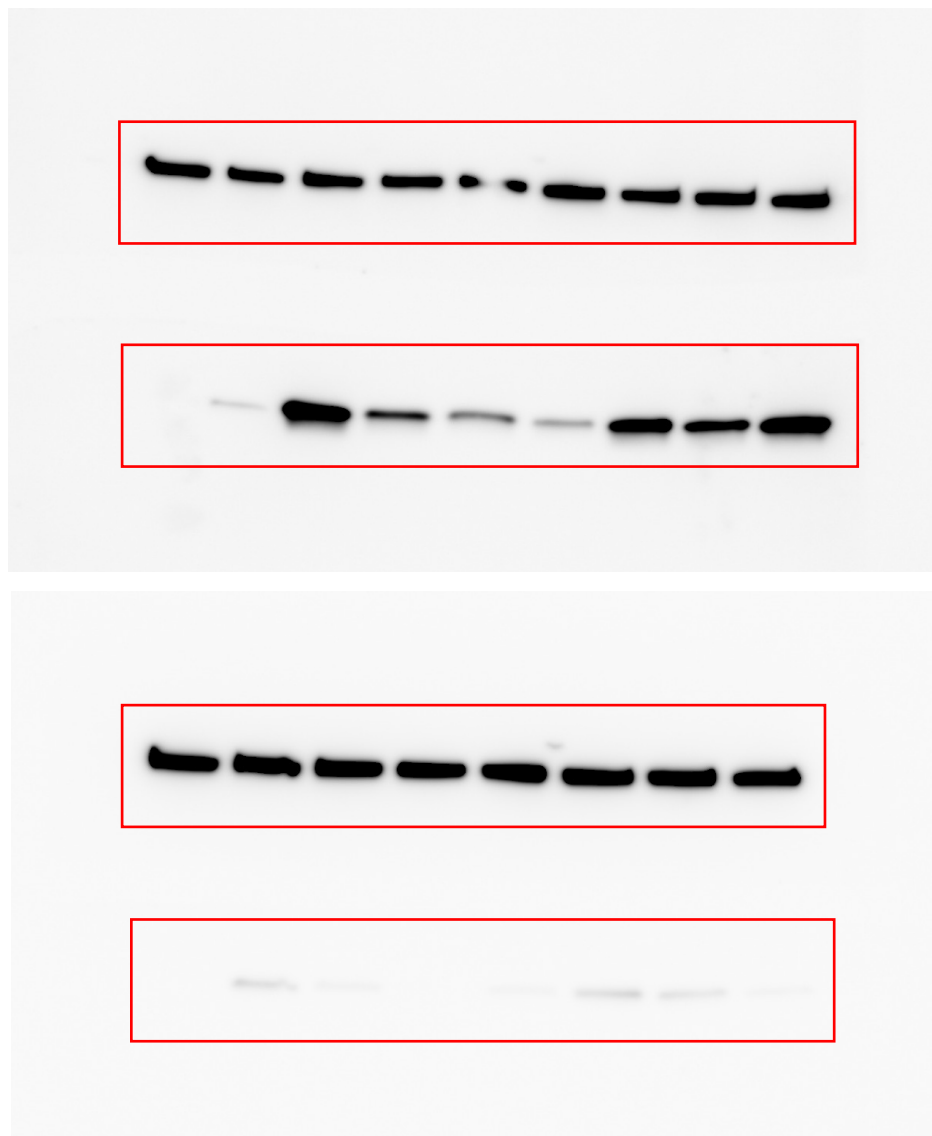

**Figure 2b**

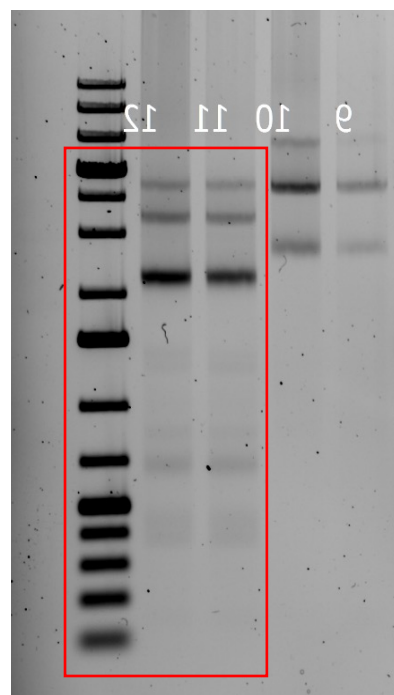

**Figure 3a**

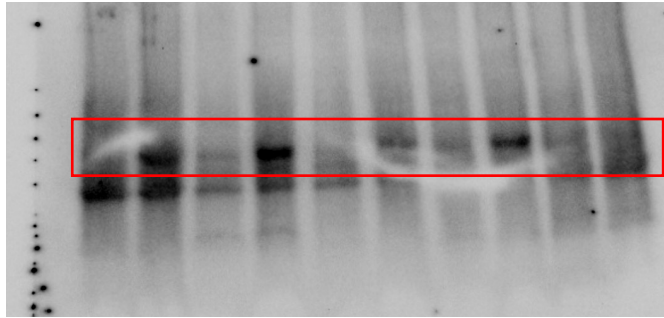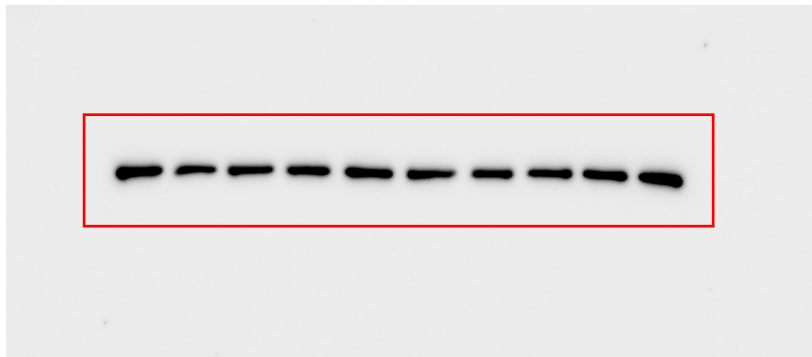

Supplement: Supplementary file 9 — Source data [file 41467_2022_33956_MOESM9_ESM.zip › Source data/uncropped WB images.pdf]
